# Effects of hydroxyapatite-coated nonwoven polyethylene/polypropylene fabric on non-mesodermal lineage-specific differentiation of human adipose-derived stem cells

**DOI:** 10.1186/s13104-020-05315-8

**Published:** 2020-10-07

**Authors:** Edward Hosea Ntege, Hiroshi Sunami, Junko Denda, Naoko Futenma, Yusuke Shimizu

**Affiliations:** 1 Department of Plastic and Reconstructive Surgery, Graduate School of Medicine, University of the Ryukyus, 207 Uehara, Nishihara, Nakagami, Okinawa, 903-0215 Japan; 2 Center for Advanced Medical Research, School of Medicine, University of the Ryukyus, 207 Uehara, Nishihara, Nakagami, Okinawa, 903-0215 Japan

**Keywords:** Adipose tissue-derived stem cells, Mesenchymal stem cells, Cardiomyocytes, Hydroxyapatite, Nonwoven scaffold, Transdifferentiation, Cell therapy

## Abstract

**Objective:**

Compared to other stem cells, the multipotency of human adipose-derived mesenchymal stem cells (ASCs) is limited. Effective approaches that trigger or enhance lineage-specific transdifferentiation are highly envisaged in the improvement of ASCs-based cell therapies. Using Immunofluorescence assays and the secretion of cardiac troponin T (cTnT) protein, we studied the impact of two substrates: Hydroxyapatite (HAp)-coated nonwoven polyethylene (PET)/polypropylene (PP) fabric and glass surfaces, representing 3 dimensional (D) and 2 D environments respectively, on the induction of cardiomyocytes – a non-mesodermal cell type from ASCs for 1–5 weeks.

**Results:**

ASCs were successfully isolated from human adipose tissue under cGMP conditions. Within 1–3 weeks, expression of cTnT in the induced 3D cultures was overall significantly higher (P < 0.021) than that in the induced 2D cultures or controls (P < 0.0009). Remarkably, after 3 weeks of culture, cTnT secretion in the induced 3D cultures gradually declined, nearly reaching levels observed in the 2D cultures. The results show that HAp-coated nonwoven PE/PP fabric could enhance lineage-specific differentiation of ASCs toward cardiac-like cells. However, the fabric might suppress growth of the transformed cells. These preliminary findings encourage further interest in validating the fabric’s potential in improving ASCs transdifferentiation.

## Introduction

ASCs -based cell therapies are highly promising in regenerative medicine [1–3]. In addition to the several important therapeutic properties [4, 5], ASCs are capable of differentiating into cells of different lineages [6, 7]. However, the potential for multilineage differentiation is limited [8]. Cardiovascular diseases (CVDs) such as ischemic heart disease and severe heart failure remain highly fatal [9, 10]. Such diseases render intense quest for novel and effective approaches to treatment using stem cells [11], and the transdifferentiation of ASCs could offer viable solutions [3]. However, several attempts to decipher and develop effective transdifferentiation systems highlight huge demand for further research [1, 12–19].

Here we hypothesized that based on previous reports [20], hydroxyapatite (HAp)-based nonwoven fabrics would play an important role in improving the transdifferentiation potential of ASCs. Therefore, we evaluated the impact of the fabrics on induction of cardiomyocytes as non-mesodermal lineage cells from ASCs using quantitative immunofluorescence analysis.

## Main text

### Materials and methods

#### Preparation of ASCs

The study design is summarized in Additional file 1, and adipose samples were obtained from participants of the previously reported ASCs clinical study (reviewed in [3]). The samples were obtained by surgeons from the Department of Plastic and Reconstruction Surgery of Ryukyu University Hospital, through a meticulous informed consent process from 5 donors aged 49 ± 1 years (range: 12–75 years) who were relatively healthy individuals. However, sample processing for ASCs: isolation, culturing, and downstream applications was random, and the donor biodata was blinded to the investigators. The ASCs isolation and initial culture procedures were based in the recently established current good manufacturing practice (cGMP)-compliant cell processing facility (CPF) [3].

##### Isolation and culturing of ASCs

Abdominal adipose samples were obtained through liposuction procedures as described elsewhere [21], and transferred to the CPF in sterile containers at 2–8 °C. Isolation of ASCs was done within 24 h using slightly modified enzyme digestion approach as described previously [3, 22]. Digestion involved the use of a prepared 1.0 WU/mL liberase MNP-S 5 mg GMP-grade solution. The solution was a blend of collagenase types I and II enzymes with medium-content thermolysin (Cat. No. 06297790001, Roche Diagnostics). Plastic adherent cells plated from stromal vascular fraction (SVF) were designated passage zero (P0) ASCs. Briefly, after 24 h incubation (5% CO_2_; 37 °C), the SVF/ASCs were washed in Dulbecco's phosphate buffered saline (DPBS; PBS, Sigma-Aldrich) and maintained in fresh serum-free STK2 medium (DS Pharma Biomedical, Osaka, Japan) supplemented with 100 IU/mL penicillin, 100 μg/mL streptomycin, and 2.5 μg/mL amphotericin every other day. Later sub-confluent adherent colonies were harvested with TrypLE™ Express (Thermo Fisher Scientific) and further cultured at a density of 5 × 10^3^ cells/cm^2^. This phase yielded P1 cultures which were harvested at 80–90% confluence for cryopreservation or further expansion and characterization.

##### Characterization of ASCs

ASCs phenotype profiling was as described previously [22]. Briefly, P3 ASCs were harvested and resuspended in PBS. A 100 μL of ASCs suspension (1 × 10^6^ cells/mL) was divided into two sets: stained and unstained (Negative control). Phycoerythrin (PE)-conjugated anti-human cluster of differentiation (CD): 29, 31, 34, 44, 45, 73, 90, and 105, and mouse IgG1, κ isotype antibodies (Biolegend, San Diego, CA, USA), and a viability dye 7-aminoactinomycin D (7-AAD) (Thermo Fisher Scientific) were utilized. All samples were assessed within 24 h of preparation using BD FACSCalibur™ flow cytometer (Becton Dickinson, San-Jose, CA, USA) and FlowJo v10 software.

ASCs differentiation potential was demonstrated through induction of osteocytes and adipocytes as described [6, 23, 24]. The existence of osteocytes and adipocytes in induced cultures was confirmed by alizarin red and oil red o staining respectively, and phase-contrast microscopy.

#### Cardiomyogenic differentiation

There were two experimental (Treatment) groups and two control (Untreated) groups: the experimental groups were subjected to media containing 5-aza-2′-deoxycytidine (5‐Aza; Sigma Aldrich) as the inducing agent, while as the control groups utilized growth media only (Negative control). This investigation did not consider a positive control at this time. Both experimental and control cultures had substrates and divided into: those cultured on cover glass (2D group), and those seeded in nonwoven fabrics (3D group) (Additional file 2). The nonwoven fabric substrates (BMK R003) were kindly supplied by Funakoshi Co., Ltd, Tokyo, Japan. The fabric was produced from polyethylene (PET) sheath/polypropylene (PP) core conjugate fibers and coated with hydroxyapatite (HAp). Preparation of the fabrics for culturing of the ASCs was performed as described elsewhere [20]. Induction of the ASCs was performed as described [25, 26]. After enzymatic harvesting, P3 ASCs were resuspended in ADSC-GM medium and seeded onto the nonwoven fabric at a density of 1 × 10^5^ cells/mL. Similar procedures were made for cultures on cover glasses. Three independent biological experiments were prepared intended for different culture monitoring periods per group, i.e., 1, 2, 3, 4, and 5 weeks. ASCs were first cultured for 24 h before replacing media with ADSC-GM supplemented with 1% insulin, human transferrin, and selenous acid (ITS) + Premix (Thermal Fisher Scientific) medium only or ITS + premix-supplemented ADSC-GM containing 10 μmol/L of 5‐Aza. The added ITS + Premix-supplemented media was again replenished after culturing for another 24 h. Thereafter, cultures were maintained in the fresh media every 3–4 days for 1–5 weeks. The morphologies of cells cultured for different periods were visualized, and images were taken under an inverted phase contrast microscope equipped with a digital camera (Olympus CKX53, Tokyo Japan).

#### Immunocytochemistry

Indirect immunofluorescence was performed following standard procedures [27]. The primary antibody for detecting cardiomyogenesis was mouse anti-rabbit cardiac troponin T (cTnT) (1:200; cat. no. ab10214; Abcam, Cambridge, UK), and the secondary antibody, Alexa Fluor™ 594 goat anti-mouse IgG (Invitrogen, Thermo fisher Scientific). 4′, 6-Diamidino-2-pheny-lindoldihydrochloride (DAPI; Sigma) was used for counterstaining. The immunofluorescently stained plates were examined by confocal laser scanning microscope (FV-1000, Olympus. Co., Tokyo, Japan) using Z-stack images with a 60 × water immersion objective. All the images were acquired with the same laser intensity and exposure time. Representative images were generated by superimposing (overlay) individual images from confocal Z-sections. The processed images were analyzed using ImageJ software (National Institutes of Health).

#### Statistics

Student’s unpaired *t*-test was used for comparisons between two experimental groups. One-way analysis of variance with Bonferroni correction for multiple comparisons was used to determine differences between mean MFI across treatment groups. All analyses were performed using GraphPad Prism, v8.0 (GraphPad Software Inc., La Jolla, CA, USA). Results are presented as mean (± SEM). Differences were considered statistically significant at P < 0.05.

### Results

#### Characterization of ASCs

The SVF/ASCs were heterogeneously shaped and included non-adherent cells. However, a week later, the P0 cells became more homogenous and displayed the distinctive spindle fibroblast-like morphology. The isolated ASCs were viable and expanded through four generations while maintaining the characteristic appearance (Additional file 3). The phenotype profile results were consistent with the of previous reports (reviewed in [3]) (Additional file 4). The differentiation potential of the isolated ASCs was also confirmed (Additional file 5).

#### ASC differentiation toward cardiomyocytes

**Fig. 1:**
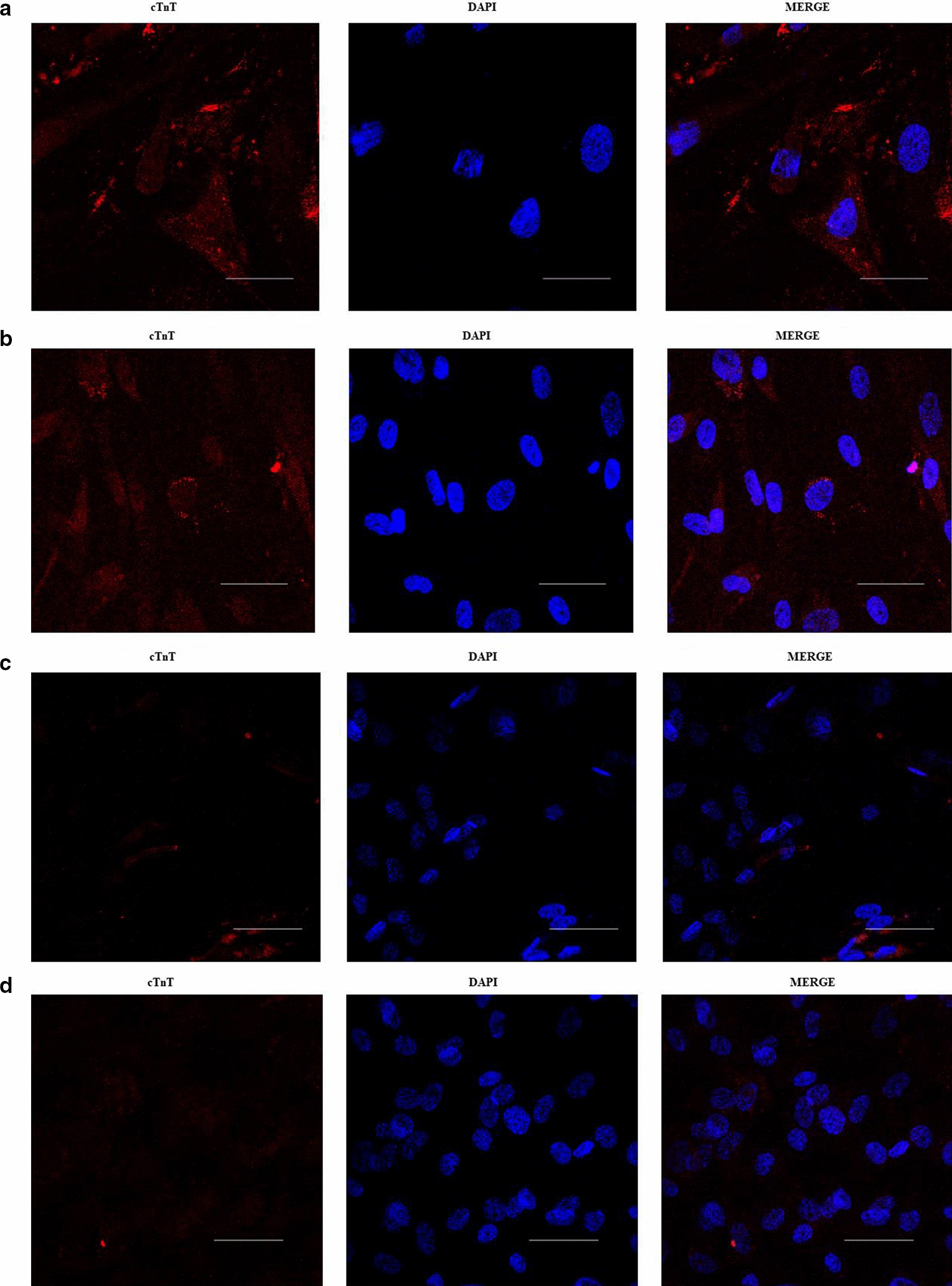

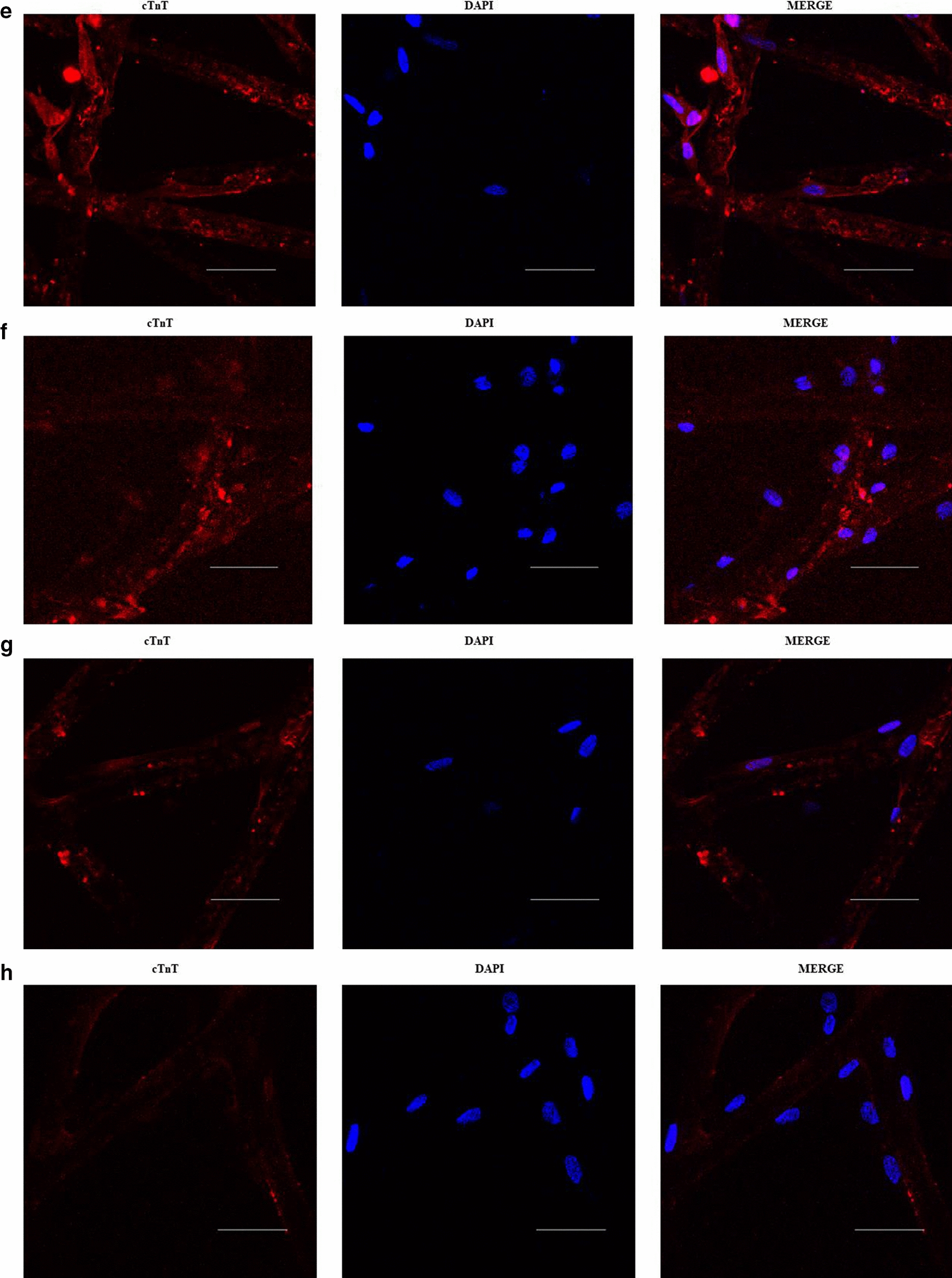
cTnT expression Immunocytochemistry Micrographs. Cell cultures were fixed with 4% paraformaldehyde and probed with mouse anti rabbit cardiac troponin T (cTnT) (red). Representative IFA images (Micrographs) of cTnT secretion in 2D and 3D cultures at 2 and 5 weeks post ITS + Premix-supplemented ADSC-GM only (untreated cultures) or ITS + Premix-supplemented ADSC-GM containing 10 μmol/L of 5‐Aza (treated cultures): **a** and **b** treated-2D cultures, **c** and **d** untreated 2D cultures (Control-GC), **e** and **f** treated-3D cultures, **g** and **h** untreated 3D cultures (control-SC). (Scale bar: 100 μm).

The observation of cells under induction with phase contrast microscopy was easier for 2D cultures than for 3D substrates (Additional file 2). Similar to previous reports, the observed changes were of gradual onset, including the transformation of most ASCs in the 5-Aza treated cultures from the characteristic fibroblast-like appearance to elongated and multinucleated cells with a tendency to globular morphology (Additional file 2a). The untransformed ASCs maintained their appearance even through repeated subcultures of the untreated groups as well. The 5-Aza treated 3D group displayed the adipocyte phenotype within and below the fabric, appeared to form aggregates, and proliferated along the fabric’s fibers. The oval-like shapes were maintained especially in the margin and middle areas of the substrate (Additional file 2b). Although the observed morphologic changes were compatible with cardiomyocyte characteristics, there were no spontaneous beating cells in the five weeks of monitoring. The confocal laser scanning fluorescence microscopy revealed cell clusters expressing cTnT proteins in the 5-aza treated cultures (Fig. 1a–h). The 5-Aza treated 3D group had fewer cells per micrograph (Table 1), but showed higher cTnT fluorescence in the round shapes along the fibers of the fabric and partially formed multicellular aggregates. In contrast, the induced 2D cultures had many almost confluent cells that grew in monolayers with a flat morphology.

**Table 1:** Immunofluorescence analysis of cardiac Troponin T expression

Micrographs	Period	No. of cells	Overall MFI	MFI/cell	MFI mean	SEM
Treated-GC	*Week 1*	24	31.404	3.02	3.413	0.248
	*Week 2*	42	52.598	3.963		
	*Week 4*	96	108.654	3.708		
	*Week 5*	97	94.06	2.961		
Treated-SC	*Week 1*	10	41.61	8.322	8.953	2.087
	*Week 2*	14	57.714	14.917		
	*Week 4*	36	78.832	7.31		
	*Week 5*	68	107.115	5.262		
Control-GC	*Week 1*^a^	–	–	–	0.7790	0.209
	*Week 2*	46	21.693	0.472		
	*Week 4*	38	43.198	1.137		
	*Week 5*	50	56.985	1.14		
Control-SC	*Week 1*	12	19.459	1.623	1.501	0.396
	*Week 2*	6	15.436	2.573		
	*Week 4*	20	17.882	0.894		
	*Week 5*	19	17.372	0.914		

*GC* cover glass, *SC* scaffold, *MFI* Mean gray (fluorescence) intensity value, *SEM* standard error of mean

^a^No measurements available for this period

**Fig. 2:**
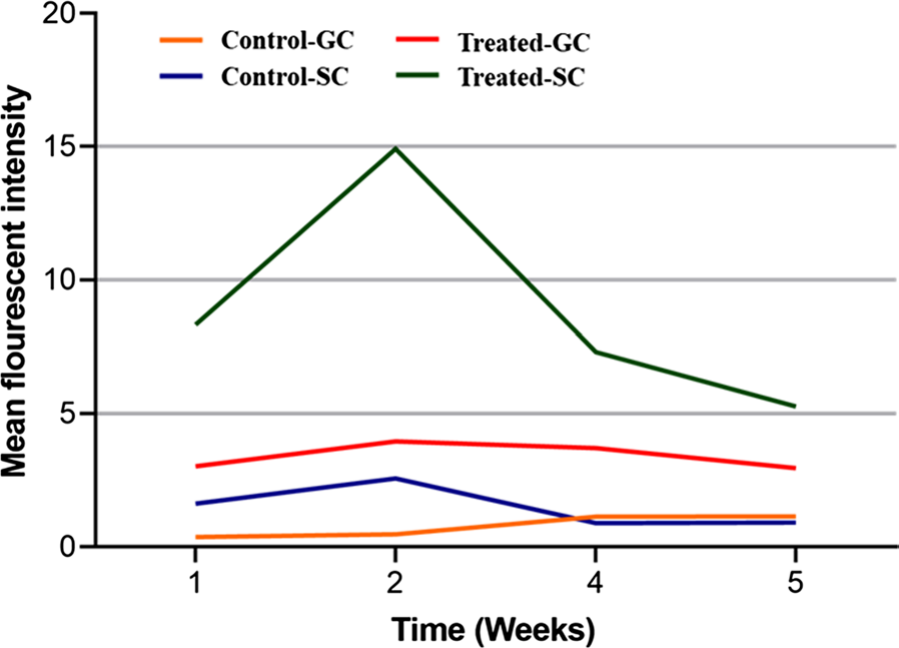
Analysis of the cTnT mean fluorescence intensities (MFI) in 2D and 3D micrographs. The line graph illustrates trends in MFI for the 2D and 3D cultures from week 1 to week 5. It presents four different groups: treated 2D cultures (Treated-GC in red color), treated 3D cultures (Treated-SC in green color), and the two control groups including the untreated 2D cultures (Control-GC in orange color) and the untreated 3D cultures (Control-SC in blue color). Overall, the MFI levels for the treated 3D cultures were exponentially higher, peaked around 2–3 weeks, and began to gradually decline over time. The MFI levels for the treated 2D cultures were slightly above that of the control cultures

The cTnT expression in 2D and 3D cultures (1–5th week) was captured as red fluorescence in the micrographs (Fig. 1a–h). The mean fluorescence intensity (mean MFI) analysis indicates cTnT secretion in induced 3D cultures (8.953 ± 2.087) was overall significantly higher than that in induced 2D cultures (3.413 ± 0.2497; P < 0.021) and the two control groups (Control-GC: 0.7790 ± 0.208; P < 0.0009, and Control-SC: 1.501 ± 0.396; P < 0.0023) (Table 1) (Additional file 6). After three weeks, the MFI levels in the induced 3D cultures decreased to nearly those of the 2D cultures and seemed to stabilize for all groups thereafter (Fig. 2).

### Discussion

ASCs could be a viable alternative source of cardiomyocytes [1, 3, 8, 28, 29]. Here we evaluated the impact of a HAp-coated nonwoven PET/PP fabric on the transdifferentiation of our cGMP-produced ASCs of a wide range of donor age (12–75 years of age), toward cardiomyocytes through immunocytochemical analysis. Although previous studies suggested that HAp-based scaffolds are useful in inducing the functional improvement of cells [20], the impact of such scaffolds on the lineage-specific differentiation potential of ASCs still remains elusive. Our preliminary findings suggest that the HAp-coated fabric could improve the transdifferentiation potential of ASCs toward cardiac-like cells, but probably does not maintain the growth of the transformed cells.

HAp is the main mineral component of mammalian hard tissues (teeth and bones) and human renal calculi [30]. Particulate HAp can have a highly porous structure, and its fibers can be electrospun and compounded with or without polymers to engineer scaffolds [20, 32]. The influence of the Hap-based fabric on the differentiation of mesodermal lineage cell types such as osteocytes has been described [31–33]. Earlier studies demonstrated enhanced differentiation, supported attachment, and proliferation, and stimulated extracellular matrix (ECM) production [31, 33]. Shu and coworkers, however, concluded that the fabric additionally suppressed growth of the transformed cells [31]. Our results show a similar trend. The cTnT levels in the induced cultures with the fabric were significantly the highest compared with those in the 2D and control cultures. The levels in 3D cultures seemed to peak at ~ 2–3 weeks, and surprisingly started to decline with time, reaching almost the same level of the induced 2D cultures. Unlike the transcription factors homeobox protein Nkx-2.5, heart- and neural crest derivatives-expressed protein 1 (HAND1), heart- and neural crest derivatives-expressed protein 2 (HAND2), and GATA 4 that are considered early cardiac protein markers, cTnT expression peaks around the 4th week. For stimulating ECM, HAp-coated scaffolds serve as a 3D microstructure and are capable of regulating various cellular processes including cell adhesion, migration, proliferation, apoptosis, and differentiation [34–36]. On the other hand, cells can exhibit different affinities for surfaces, and different surfaces can adsorb similar and unique ECM proteins that can suppress their growth [31]. The growth of the induced ASCs cultured on the HAp-coated nonwoven PE/PP fabric might have suffered a similar fate, hence the dramatic fall in the cTnT levels within a short time.

Some reports suggest poor induction of ASCs toward cardiac-like cells by 5-Aza [27, 36, 37]. The use of 5-Aza could as well have contributed to the study’s observations, including the lack of spontaneously beating cells. As previously described [38], the effectiveness of 5-Aza depends on hypomethylating levels of the cells, and DNA methylation is important for the preservation of pluripotency and self-renewal of stem cells during cardiac cell differentiation. However, methylation of adipocytes can be influenced specially by donor variations in metabolic state by the age, sex, weigh, exercise, fat distribution, and glucose homeostasis [3, 39, 40].

### Conclusion

We assessed our cGMP-produced ASCs and cardiac-like cells derived thereof in this study. We successfully reproduced earlier findings on the ability of our ASCs to (i) thrive within the designed culture conditions, (ii) display MSC biological properties including surface markers, and (iii) transform and display phenotypes of mesodermal and non-mesodermal lineages, while evaluating the effect of a HAp-based substrate on the cells’ transdifferentiation potential. The results showed that the Hap-based fabric could improve in vitro transdifferentiation of ASCs.

## Limitations

This study was limited to microscopy and immunocytochemistry observations for a single cardiac marker and utilized a small sample size. In addition, unimproved HAp-based nonwoven fabrics were used without in-depth characterization. Further validation of this investigation with positive controls and with a series of other biochemical, biophysical, and cell-based assays is recommended.

## Supplementary information

**Additional file 1.** A schema of the experimental design. The methodology was as described elsewhere [27, 28]. ASCs were cultured until P3, assessed for cell viability, proliferation, MSC phenotype and multilineage potential. Then, the P3 ASCs were seeded at a similar density of 1 × 10^5^ cells/mL in 1 mL of ADSC-GM medium on to the 2D and 3D platforms and cultured overnight. After 24 h, medium was replaced with ADSC-GM supplemented with 1% ITS + Premix and 10 μmol/L of 5‐Aza or ADSC-GM supplemented with 1% ITS + Premix only for the control experiments and cultured for another 24-h period (Day 0–1). Thereafter, cultures were washed 3X with PBS and maintained in fresh ITS + Premix-supplemented media every 3–4 days for 1–5 weeks. Cells were harvested weekly for cardiac phenotype monitoring by phase contrast microscopy and indirect immunocytochemical analysis (IFA).

**Additional file 2.** Morphological observations with the phase-contrast microscopy (PDF file format). (a) Morphological changes of the induced ASCs in 2D cultures: (i) undifferentiated ASCs with fibroblast-like appearance (control), (ii) 2 weeks of induced cultures showing multinucleation and elongation, (iii) Four weeks of induced cultures showing oval-like, fork-like and myotube-like structures. (b) Morphological observations of the induced ASCs in 3D cultures: (i) HAp-coated nonwoven PET/PP fabric, × 20 magnification, (ii) uninduced ASC culture with fibroblast-like appearance below the nonwoven fibers (control), (iii) representative of the 3D culture phase-contrast microscopy images, where cell morphology was relatively difficult to describe.

**Additional file 3.** Phase-contrast microscopic observations of the ASCs. From (i) passage 1, (ii) passage 2 to (iii) passage 3 showing increasing homogeneity and the distinctive spindle fibroblast-like morphology in culture (20 × objective; Scale bar, 100 μm).

**Additional file 4.** ASCs phenotype profiling. ASC surface markers as determined by flowcytometry analysis in P3 ASC cultures for (i)–(ix): Phycoerythrin (PE), CD105, CD73, CD44, CD29, CD90, CD45, CD34, and CD31 respectively. PS; percentage of positive cells.

**Additional file 5.** Assessment of ASCs multilineage potential. (i) undifferentiated cells (control), (ii) Osteocyte differentiation confirmed using Alizarin staining for the presence of calcium deposition in the cells, (iii) Adipogenic lineage confirmed by oil red O staining for the presence of lipid granules in the differentiated cells. (Scale bar, 100 μm).

**Additional file 6.** Histogram showing the statistical analysis of cTnT MFI in 2D and 3D cultures. Graphical representation of MFI levels in 2D and 3D cultures corresponding to the 5-aza-2′-deoxycytidine induced ASCs (Treated-GC and Treated-SC respectively), and the two untreated control groups: Control-GC and Control-SC respectively. Pairwise comparison showed that mean MFI in Treated-GC against Treated-SC, Treated-SC against Control-GC, and Treated-SC against Control-SC groups were significantly different after correction for multiple comparisons using the Bonferroni test (P < 0.05).

## Data Availability

The data used to support the findings of this study are available from the corresponding author upon request.

## Abbreviations

ADSC-GM: Human adipose derived stem cells- Growth Media
ASCs: Adipose tissue-derived stem cells
CD: Cluster of differentiation
cGMP: Current Good Manufacturing Practice
CPF: Cell Processing Facility
cTnT: Cardiac contractile proteins (cTnT)
DAPI: 4′,6-Diamidino-2-phenylindole,
DPBS; PBS: Dulbecco’s phosphate buffered saline
ECM: Extracellular Matrix
GATA4: GATA Binding Protein 4
HAND1: Heart- and neural crest derivatives-expressed protein 1 (HAND1)
HAND2: Heart- and neural crest derivatives-expressed protein 2 (HAND2)
Hap: Hydroxyapatite
MFI: Mean fluorescence intensity
Nkx2.5: Homeobox protein Nkx-2.5
PET: Polyethylene
PP: Polypropylene
PE: Phycoerythrin
7-AAD: 7-Aminoactinomycin D
