# Supplementary figures and images for: Effects of hydroxyapatite-coated nonwoven polyethylene/polypropylene fabric on non-mesodermal lineage-specific differentiation of human adipose-derived stem cells
Source: BMC Res Notes. 2020 Oct 7;13:471. doi: 10.1186/s13104-020-05315-8 (PMC7542906; doi:10.1186/s13104-020-05315-8)

Additional file 2a

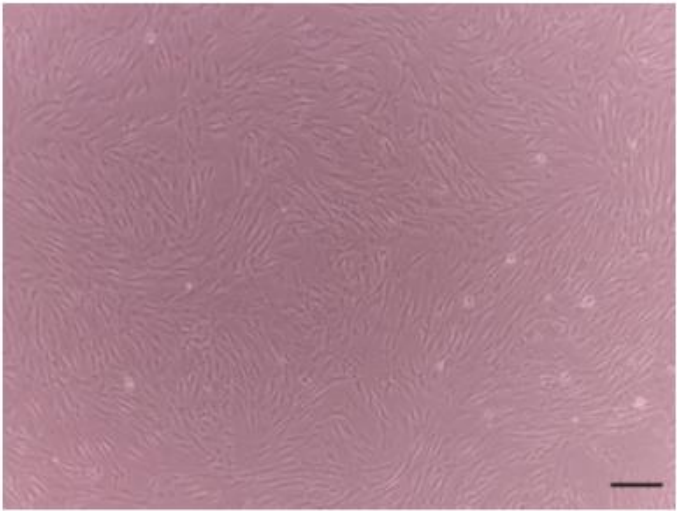

(i)

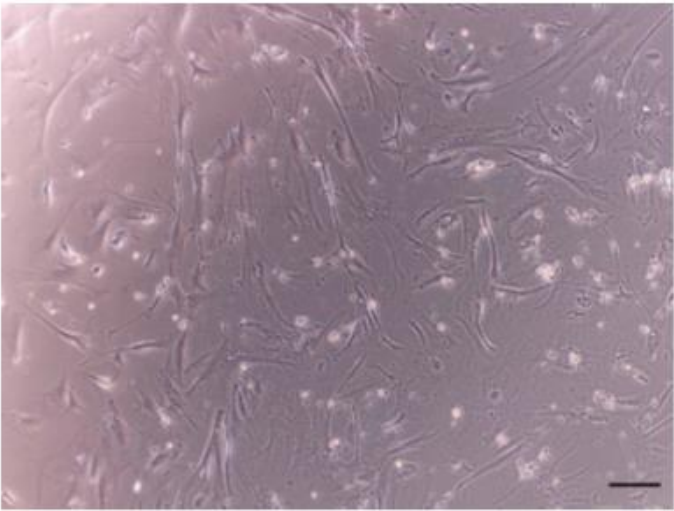

(ii)

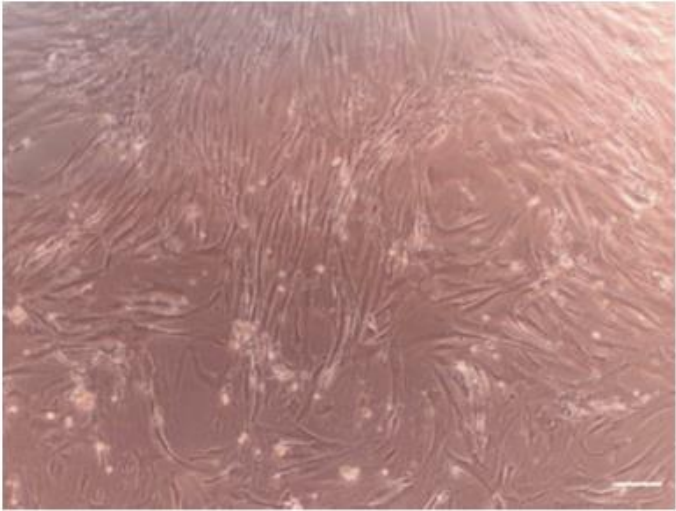

(iii)

Additional file 2b

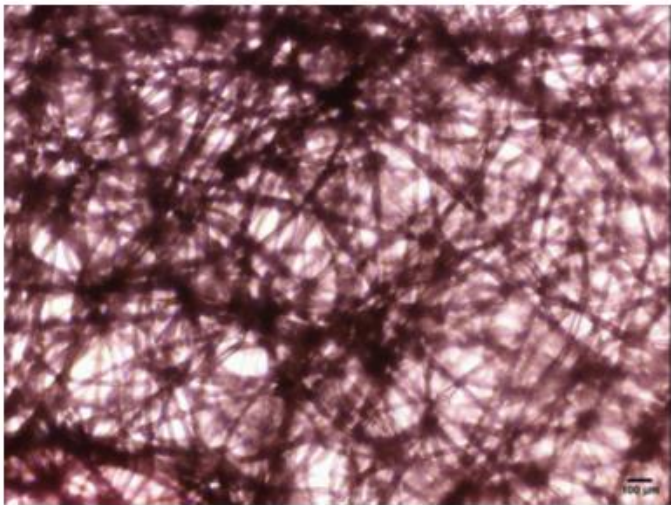

(i)

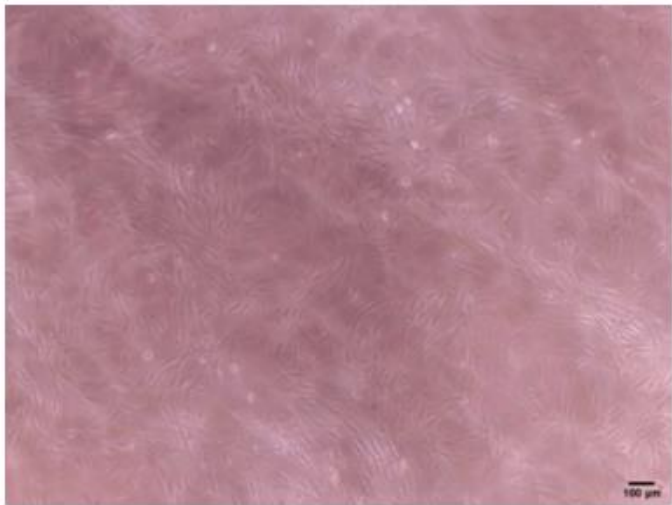

(ii)

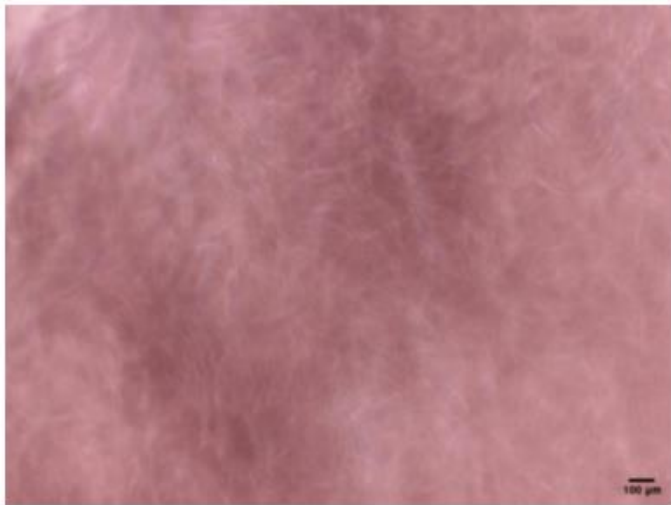

(iii)

Supplement: Supplementary file 2 — Additional file 2. Morphological observations with the phase-contrast microscopy (PDF file format). (a) Morphological changes of the induced ASCs in 2D cultures: (i) undifferentiated ASCs with fibroblast-like appearance (control), (ii) 2 weeks of induced cultures showing multinucleation and elongation, (iii) Four weeks of induced cultures showing oval-like, fork-like and myotube-like structures. (b) Morphological observations of the induced ASCs in 3D cultures: (i) HAp-coated nonwoven PET/PP fabric, × 20 magnification, (ii) uninduced ASC culture with fibroblast-like appearance below the nonwoven fibers (control), (iii) representative of the 3D culture phase-contrast microscopy images, where cell morphology was relatively difficult to describe. [file 13104_2020_5315_MOESM2_ESM.pdf]

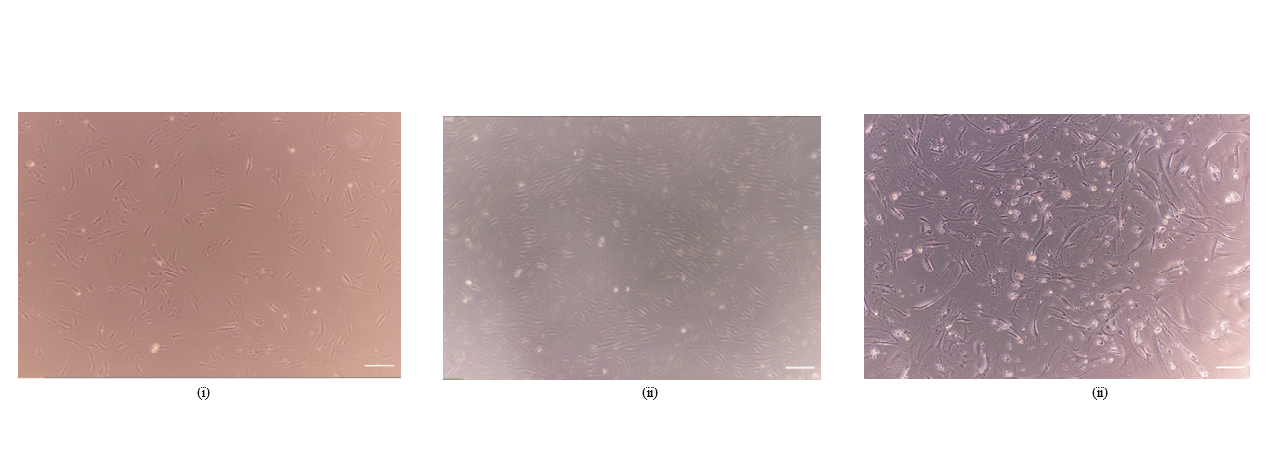

Supplement: Supplementary file 3 — Additional file 3. Phase-contrast microscopic observations of the ASCs. From (i) passage 1, (ii) passage 2 to (iii) passage 3 showing increasing homogeneity and the distinctive spindle fibroblast-like morphology in culture (20 × objective; Scale bar, 100 μm). [file 13104_2020_5315_MOESM3_ESM.tif]

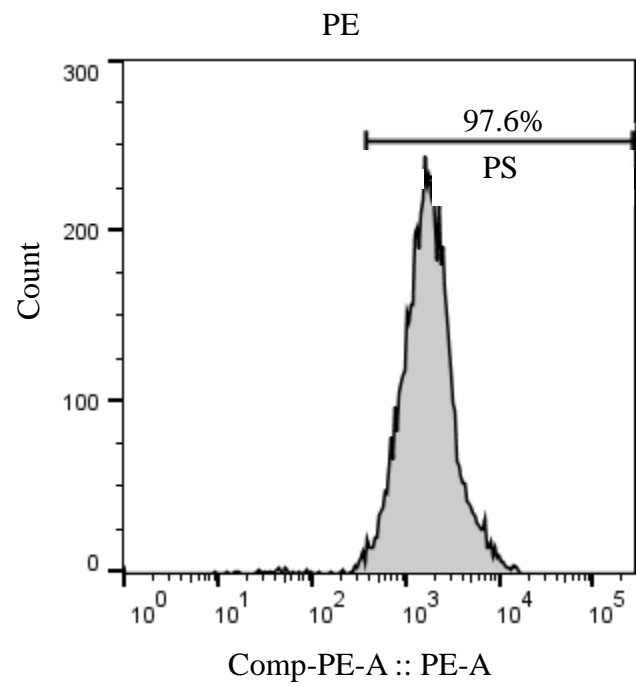

(i)

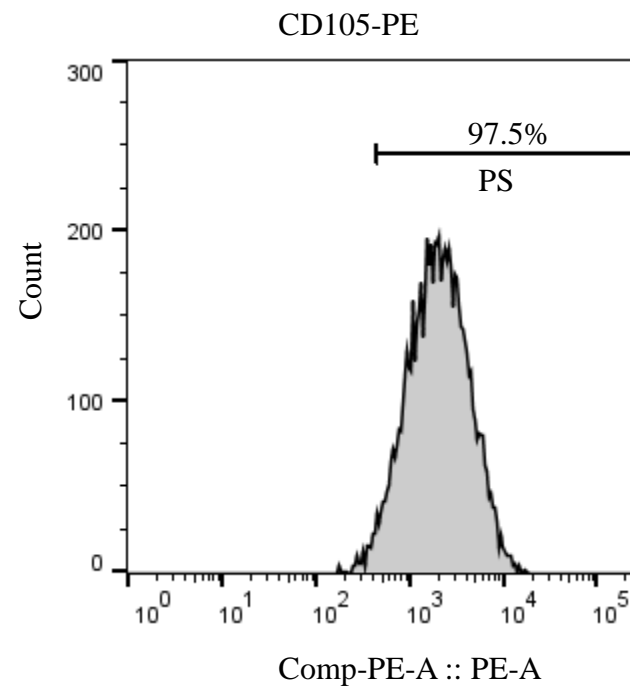

(ii)

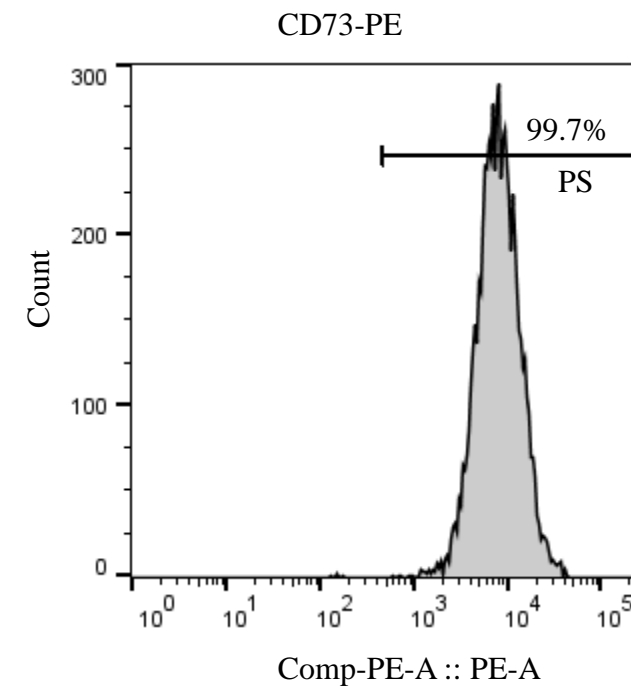

(iii)

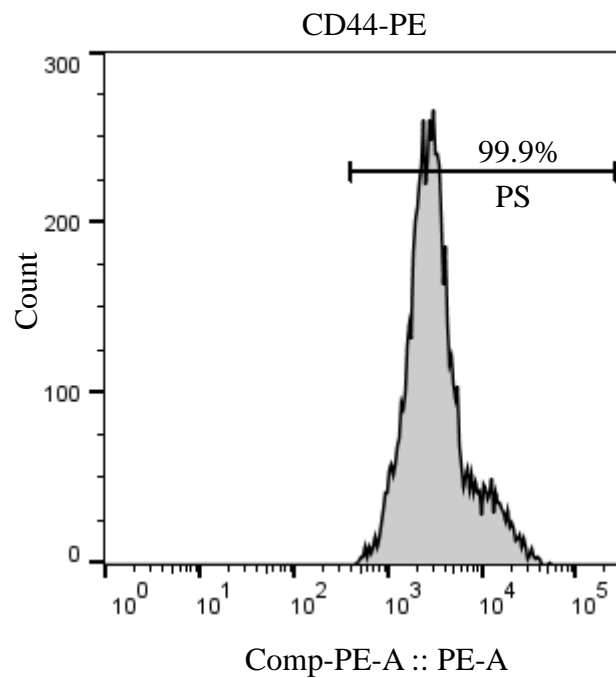

(iv)

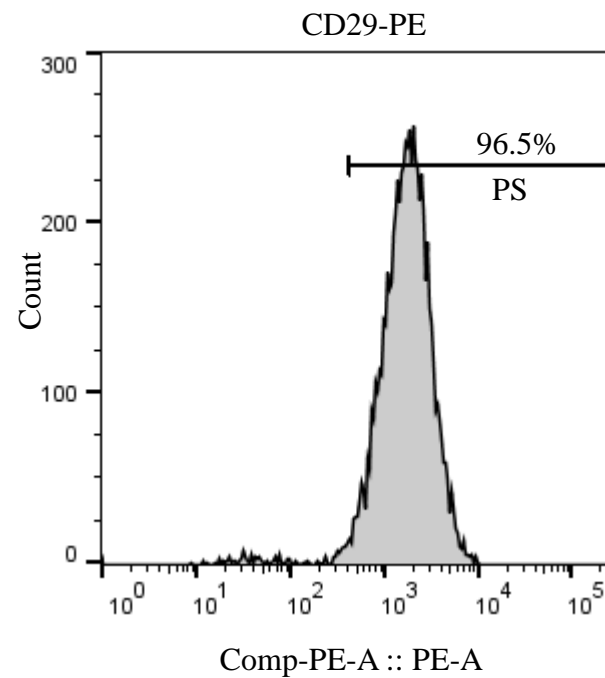

(v)

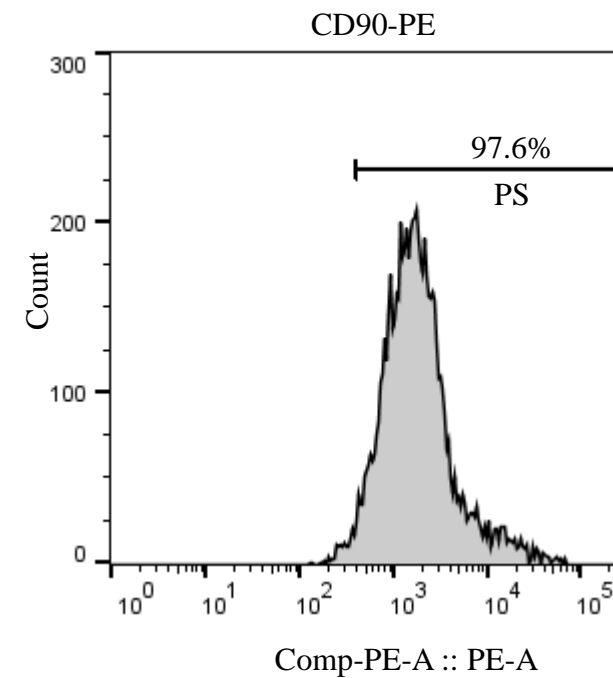

(vi)

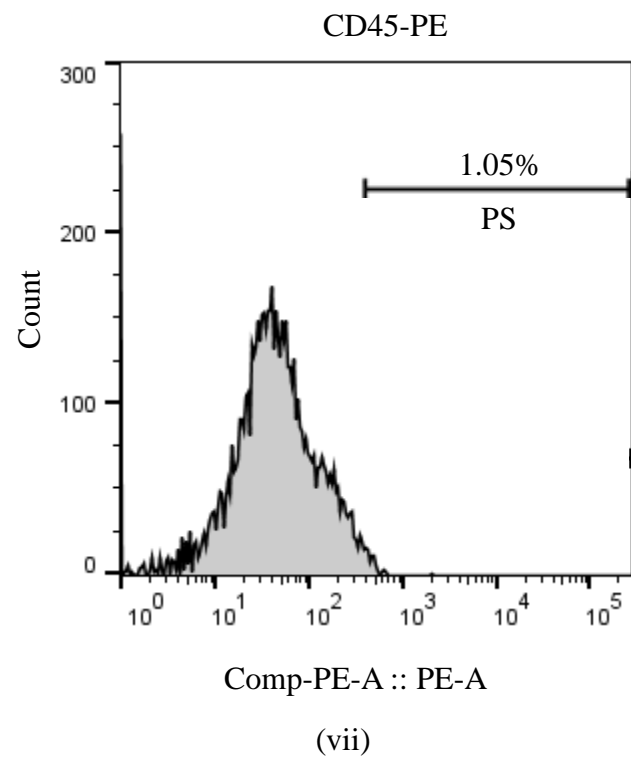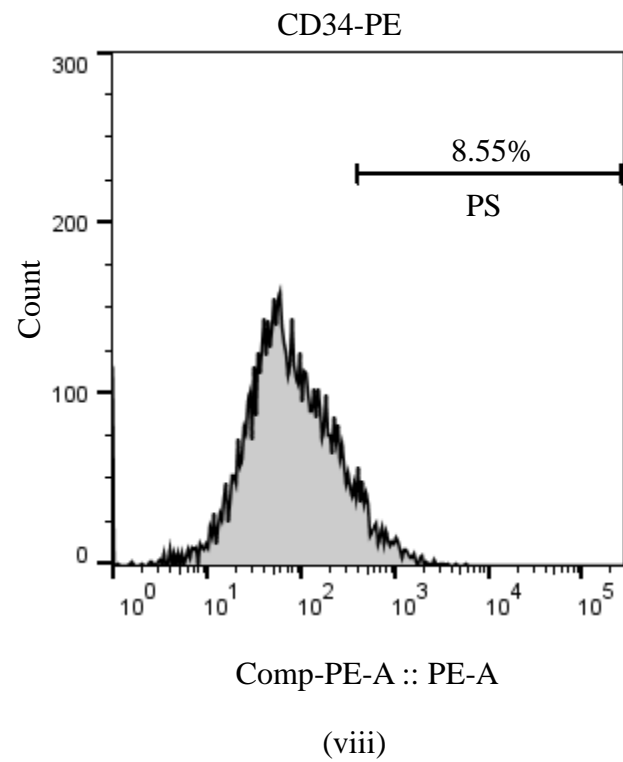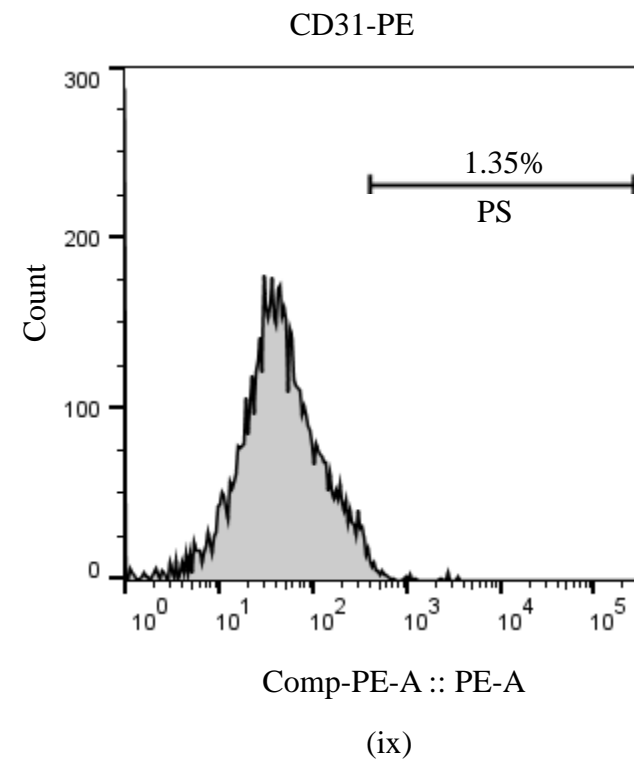

Supplement: Supplementary file 4 — Additional file 4. ASCs phenotype profiling. ASC surface markers as determined by flowcytometry analysis in P3 ASC cultures for (i)–(ix): Phycoerythrin (PE), CD105, CD73, CD44, CD29, CD90, CD45, CD34, and CD31 respectively. PS; percentage of positive cells. [file 13104_2020_5315_MOESM4_ESM.pdf]

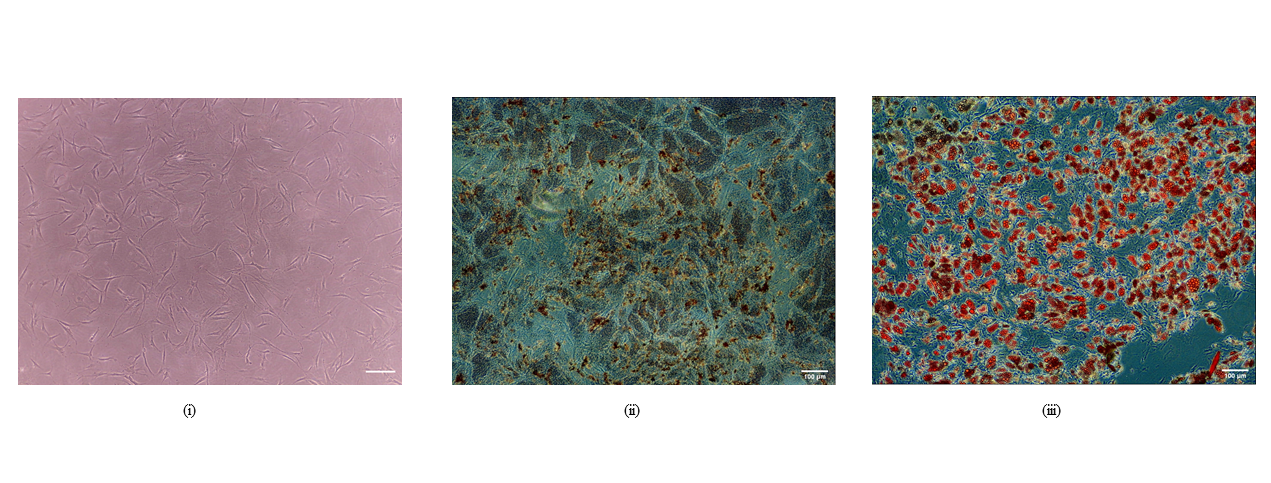

Supplement: Supplementary file 5 — Additional file 5. Assessment of ASCs multilineage potential. (i) undifferentiated cells (control), (ii) Osteocyte differentiation confirmed using Alizarin staining for the presence of calcium deposition in the cells, (iii) Adipogenic lineage confirmed by oil red O staining for the presence of lipid granules in the differentiated cells. (Scale bar, 100 μm). [file 13104_2020_5315_MOESM5_ESM.tif]

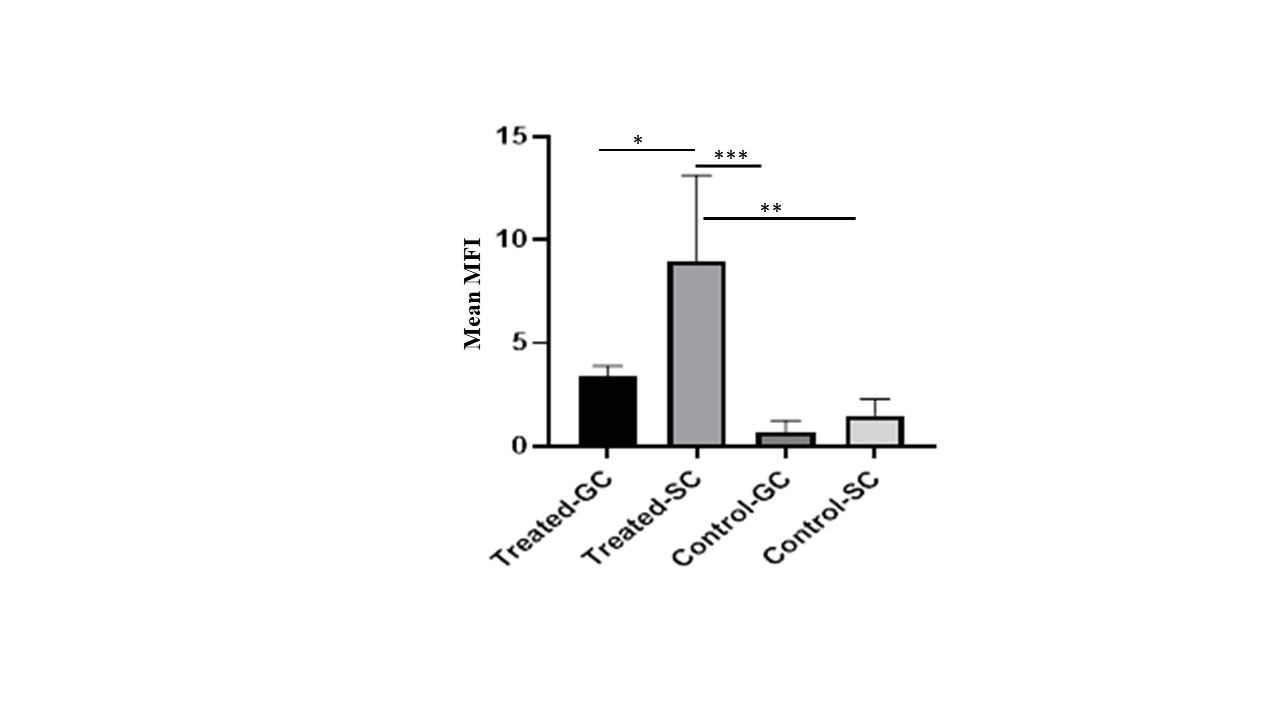

Supplement: Supplementary file 6 — Additional file 6. Histogram showing the statistical analysis of cTnT MFI in 2D and 3D cultures. Graphical representation of MFI levels in 2D and 3D cultures corresponding to the 5-aza-2′-deoxycytidine induced ASCs (Treated-GC and Treated-SC respectively), and the two untreated control groups: Control-GC and Control-SC respectively. Pairwise comparison showed that mean MFI in Treated-GC against Treated-SC, Treated-SC against Control-GC, and Treated-SC against Control-SC groups were significantly different after correction for multiple comparisons using the Bonferroni test (P < 0.05). [file 13104_2020_5315_MOESM6_ESM.tif]
